# Flubendazole inhibits PD-1 and suppresses melanoma growth in immunocompetent mice

**DOI:** 10.1186/s12967-023-04289-y

**Published:** 2023-07-14

**Authors:** Yue Li, Ben Wu, Md Jakir Hossain, Lily Quagliata, Connor O’Meara, Marc R. Wilkins, Susan Corley, Levon M. Khachigian

**Affiliations:** 1 Vascular Biology and Translational Research, Department of Pathology, School of Biomedical Sciences, Faculty of Medicine and Health, University of New South Wales, Sydney, NSW 2052 Australia; 2 Department of Otorhinolaryngology, Head & Neck Surgery, Prince of Wales Hospital, Randwick, NSW 2031 Australia; 3 Systems Biology Initiative, Ramaciotti Centre for Genomics, University of New South Wales, Sydney, NSW 2052 Australia

**Keywords:** Flubendazole, Benzimidazole, Melanoma, Programmed cell death protein-1

## Abstract

**Background:**

Immune checkpoint inhibitor therapy has revolutionized the clinical management of a diverse range of cancer types, including advanced cutaneous melanoma. While immunotherapy targeting the PD-1/PD-L1 system has become standard of care, overall response rates remain unsatisfactory for most patients and there are no approved small molecule inhibitors of the PD-1/PD-L1 system. Flubendazole (FLU) is an anthelmintic that has been used to treat worm infections in humans and animals for decades.

**Methods:**

Here we tested the anti-cancer activity of systemically delivered FLU with suppression of PD-1 in immunocompetent mice.

**Results:**

In C57BL/6J mice bearing subcutaneous B16F10 melanoma, FLU reduced both tumor growth and PD-1 protein levels without affecting levels of PD-L1. FLU’s suppression of PD-1 was accompanied by increased CD3^+^ T cell infiltration. Western blotting with extracts from human Jurkat T cells showed that FLU inhibited PD-1 protein expression, findings confirmed by flow cytometry. To gain mechanistic insights on FLU’s ability to suppress PD-1 protein levels, we performed bulk RNA sequencing on extracts of Jurkat T cells exposed to the benzimidazole for 4 h. From a pool of 14,475 genes there were 1218 differentially-expressed genes; 687 with increased expression and 531 with decreased expression. Among the genes induced by FLU was the AP-1 family member, *JUN* and surprisingly, *pdcd1*. KEGG pathway analysis showed FLU up-regulated genes over-represented in multiple pathways (p < 0.01), the top hit being amoebiasis. FLU also affected the expression of genes in cancer-associated pathways, both through down-regulation and up-regulation. Gene set enrichment analysis revealed a large number of immunological signature gene sets correlated with FLU treatment, including gene sets associated with T cell differentiation, proliferation and function. The AP-1 inhibitor T5224 rescued PD-1 protein expression from inhibition by FLU.

**Conclusion:**

This study is the first to show that FLU can inhibit melanoma growth with PD-1 suppression in immunocompetent mice.

**Supplementary Information:**

The online version contains supplementary material available at 10.1186/s12967-023-04289-y.

## Introduction

Immune checkpoint therapy has radically changed the management of a diverse range of cancer types, including advanced cutaneous melanoma [1]. For example, antibodies to programmed cell death protein 1 (PD-1) (such as nivolumab/Opdivo, pembrolizumab/Keytruda and cemiplimab/Libtayo), PD-L1 (atezolizumab/Tecentriq, avelumab/Bavencio and durvalumab/Imfinzi) and cytotoxic T-lymphocyte–associated antigen 4 (CTLA-4) (ipilimumab/Yervoy) have become standard of care in tumor immunotherapy. However, around 1 in 2 patients do not respond to PD-1 antibody-based therapies or the durability of this response is not sustained and immune-related adverse events are common [2–5]. There are no approved small molecule inhibitors of PD-1, PD-L1 or CTLA-4 that could be used as alternatives to antibodies. Small molecules offer potential advantages over antibodies such as greater stability, medicinal chemistry, low cost, penetrant effects across whole cell populations, intracellular signalling pathway targeting and potential oral administration meaning avoidance of in-clinic administration and reduced health costs [6].

Drug repurposing, the process by which older medicines can be redeveloped for new therapeutic indications offers a range of advantages over conventional drug discovery programs [7]. Repurposing can develop de-risked medicines with known pharmacology, tolerance and toxicity at potential lower cost. A recent study found that the median capitalized R&D cost of bringing a new drug to market was approximately $985 million and > 8 years per drug [8]. A well-known example of a repurposed drug is sildenafil, which was originally developed in the context of angina and is widely used for erectile dysfunction [9]. A more recent example is remdesivir which was developed for Ebola virus and is used for the management of SARS-CoV-2 infection [10].

PD-1 (CD279), encoded by the *pdcd1* gene and first discovered in 1992 [11], is an inhibitory receptor expressed by T and B cells, natural killer cells and certain myeloid cell populations [12]. We and others have shown that PD-1 is also expressed by melanoma cells [13, 14]. PD-1, a type I transmembrane glycoprotein bound by glycoproteins PD-L1 (B7-H1, CD274) and PD-L2 (B7-DC, CD273) [15, 16] can promote tumor growth by preventing activation of cytotoxic T-cells [13, 14]. Given current limitations of antibody-based therapies, small molecule alternatives may provide an alternative means to disrupt the PD-1/PD-L1 axis. For example, Wang et al. recently reported their discovery of compound 17 which inhibits the PD-1/PD-L1 interaction and results in degradation of PD-L1 [17]. Studies in our laboratory show that flubendazole (FLU), a benzimidazole used as an anthelmintic for over 40 years [18], can inhibit human melanoma growth and metastasis in immunodeficient mice [14]. Others have also shown that FLU can cause mitotic catastrophe [19], disrupt cell cycle progression [20] and promote ferroptosis [21]. However, it is unclear whether FLU can affect PD-1 expression in T cells or influence the growth of melanoma in immunocompetent mice. In this paper we show that FLU can inhibit melanoma growth and PD-1 expression in immunocompetent mice and that FLU’s inhibition of PD-1 involves the transcription factor AP-1.

## Materials and methods

### Mouse tumor experiments

Six to eight week old female C57BL/6 J mice were sourced from Animal Resources Centre (Perth). Animals were inoculated subcutaneously with B16F10 cells (1 × 10^5^ cells/animal in 100 µl DMEM containing 10% FBS and 50% Matrigel) into the shaven right flank on Day 0. These cells were obtained from ATCC. Mice were randomized on Day 5 into groups with mean tumor volume 40–45 mm^3^ per group. Treatments were started on Day 7, vehicle or FLU (Advanced Molecular Technologies, Scoresby) was administered intraperitoneally (20 ml/kg or 200 mg/kg, i.p.) once daily on a 5 days-on/2 days-off schedule. FLU was suspended at 10 mg/ml in vehicle (saline (0.9% NaCl) with 0.5% Tween 80 and 0.01% DMSO) and sonicated. Animals were weighed daily after Day 5. The general health condition and attitude of each animal was monitored daily throughout the study. Tumors were measured by length, height and width in millimeters daily. Tumor volumes were calculated using formula V = L × H × W × π/6. If a second tumor occurred in an animal, both tumor volumes were measured and their volumes were combined. After euthanasia by isoflurane overdose and cervical dislocation, tumors of each animal were fixed in 10% neutral buffered formalin. The protocol was approved by the UNSW Animal Care and Ethics Committee.

### Cell culture, Western blotting and flow cytometry

Human Jurkat T cells grown in RPMI 1640 with, pH 7.4, containing 10% FBS and penicillin/streptomycin were seeded into 6 well plates and treated with FLU or vehicle for the indicated times. Where indicated, T5224 (cat. S8966, Selleck Chem) or vehicle was added 2 h prior to the addition of FLU. Total cell extracts were prepared in RIPA buffer and lysates were resolved by SDS-PAGE and Western blot analysis with primary antibodies target PD-1 (1:1000, cat. ab214421, Abcam; 1:500, cat. 86163, CST, which was used for the T5224 studies), JUN (1:500, cat. ab32137; Abcam), FOS (1:500, cat. no. 2250; CST); or β-actin (1:3000, cat. A5316, Sigma) and followed by chemiluminescence detection using the Western Lightning Chemiluminescence system (ThermoFisher, cat. A38556) and visualized with an ImageQuant^™^ LAS 4000 Biomolecular Imager.

Alternatively, cells were washed and resuspended (5 × 10^6^ cells/ml) in Stain Buffer. One hundred µl was added to 12 × 75 mm tubes and 5 µl of BV421 conjugated mouse anti-human CD279 (PD-1) (1:20, BD, cat. 562,516) or BV421 conjugated mouse IgG_1_ (1:20, BD, cat. 562,438) was added and then incubated for 45 min at 22 °C, protected from light. Cells were washed twice with 2 ml of Stain Buffer, centrifuged and pellets were resuspended in 0.5 ml Stain Buffer. Stained cell suspensions were analysed by flow cytometry using a BD LSR Fortessa X20.

### Immunohistochemical processing and staining

Formalin-fixed, paraffin embedded sections were prepared from tumors. Heat-induced epitope retrieval was applied to all deparaffinized Sects. (4 μm Superfrost slides) with citrate buffer, pH 6.0 for 5 min at 110 °C. Sections were blocked with endogenous AP (levamisole) blocking agent (DAKO, S2003) for 10 min and then with 2% skim milk for 20 min. Rabbit monoclonal anti-PD-1 (cat. ab214421) and rabbit monoclonal anti-CD3 antibody (SP7) (cat. ab16669) were obtained from Abcam. Rabbit polyclonal anti-PD-L1 (cat. PA5-20,343) antibodies were obtained from ThermoFisher. Slides were incubated with primary antibody for 1 h at room temperature and then for 10 min with MACH3 Rabbit AP-Polymer Detection solution (probe incubation) (Biocare Medical, M3R533 G, H, L). After rinsing with buffer, the slides were incubated with MACH3 Rabbit AP-Polymer Detection solution (polymer incubation) (Biocare Medical, M3R533 G, H, L) for a further 10 min. Slides were incubated with red chromogen (Warp RedTM Chromogen Kit) for 5 min and counterstained in haematoxylin and Scott blue. Slides were dried with filter paper and dehydrated in xylene then coverslipped.

Immunostained slides were scanned using an Aperio ScanScope XT slide scanner (Leica Biosystems, Mt Waverley, Vic, Australia) and images were captured using ImageScope software (Leica Biosystems). Tissue area (µm^2^) and integrated optical density (IOD) of positive staining (red chromogen) using Image-Pro Plus software (Cybernetics, Bethesda, MD, USA). Positive staining cells numbers were manually counted for CD3 using Image-Pro Plus software (Cybernetics, Bethesda, MD, USA).

### RNA-seq and bioinformatics analysis

Jurkat T cells grown to confluence in 100 mm plates in complete medium were incubated with 10 µM FLU or vehicle for 4 h. Total RNA was extracted using the RNeasy Mini Kit (Qiagen, cat. 74,004) with minor modification. Briefly, the cells were washed twice with cold 1 × PBS and TRIzol reagent (ThermoFisher, cat. 15,596,026) was used to lyse the cells. Chloroform was added to the mixture prior to microfuge centrifugation at 13,000 rpm for 15 min at 4 °C. The upper aqueous layer (containing total RNA) was transferred to microtubes, isopropanol was added and loaded into RNeasy columns. Columns were washed with buffers RPE and RW1. Total RNA was eluted using ribonuclease-free water. Six samples (3 biological replicates of each condition) were submitted to the UNSW Ramaciotti Centre for Genomics for TruSeq Stranded mRNA-seq preparation and sequencing by NextSeq 6000 to produce 75 bp single end reads.

RNA-seq reads were assessed for quality using FastQC (v0.11.8) www.bioinformatics.babraham.ac.uk/projects/fastqc/). Subread was used for aligning reads to the Ensembl human genome (GRCh38) and then the featureCounts function of Subread was used to quantify reads (http://subread.sourceforge.net/). Reads assigned to gene features ranged from 46.6 M to 53.8 M reads per sample. The R packages edgeR and limma (voom) [22] were used to identify differentially-expressed genes (DEG) comparing the FLU treated Jurkat T cells with vehicle-treated Jurkat T cells. Lowly expressed genes were filtered out leaving those genes with at least 1 count per million (CPM) in at least 3 samples (n = 14,475) for further analysis. The functions lmFit, eBayes and treat were used to identify genes which differed in the FLU treated cells with a least a log2 (1.2) fold change. Significantly DEG were taken as having an adjusted p < 0.05. R plotting functions including plotMDS (limma) and pheatmap R package were used to prepare plots. The kegga function (limma) was used to perform KEGG pathway analysis.

### Gene set enrichment analysis (GSEA)

Gene counts for 27,729 genes with a symbol were normalized using the TMM function of edgeR [23] and used as input into the desktop version of GSEA (v4.3.2, Broad Institute). GSEA then generated a ranked list and analyzed the list to ascertain the degree to which a gene set was overrepresented at the top or bottom of the list, thus generating a normalized enrichment score (NES) for each gene set. Gene set analysis was run using the C7 immunologic signature gene set collection and C2 curated gene sets from MSigDb. Gene set enrichment was ranked by NES and gene sets with a false discovery rate (FDR) < 0.25 were considered significant.

### Statistics

Analysis was performed using Graphpad PRISM v9. If distribution was not normal, Mann–Whitney or Kruskal–Wallis test was performed. Normally distributed data was analyzed by t test or one-way ANOVA. Plotted data represent mean ± SEM. Differences were considered significant when p ≤ 0.05. Where indicated, *p ≤ 0.05, **p < 0.01, ***p < 0.001, ****p < 0.0001.

## Results

### *FLU suppresses melanoma growth in immunocompetent mice*

**Fig. 1:**
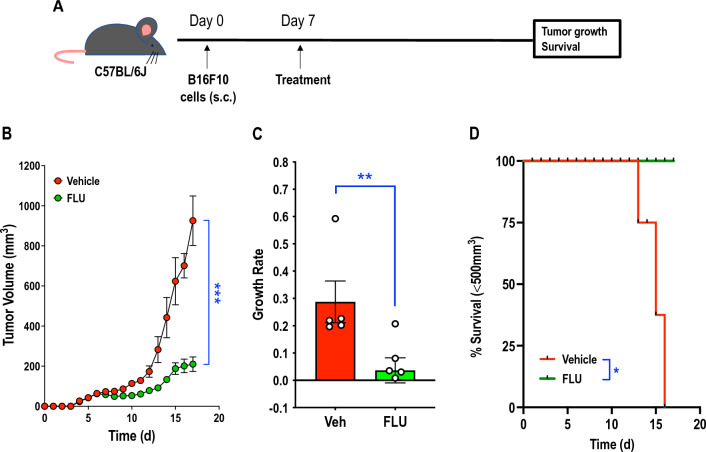
FLU suppresses melanoma growth in immunocompetent mice. **A** Schedule of mouse treatments. **B** C57BL/6J mice bearing s.c. B16F10 tumors were administered with FLU or vehicle (200 mg/kg or 20 ml/kg, respectively, i.p.) once daily on a 5 days-on/2 days-off schedule. Treatment commenced on Day 7. Tissues were collected for further analysis on Day 17. Data represent mean ± SEM. n = 6 mice/group. Statistical significance was assessed by t test. ***p < 0.001. **C** Tumor growth rates were determined by calculating the natural log (ln) of individual tumor volumes on Days 15–17 and calculating slopes in MS Excel. Data represent mean ± SEM. n = 5–6 mice/group. Statistical significance was assessed by Mann Whitney test. **D** Kaplan–Meier survival analysis was performed with a tumor size limit of 500 mm^3^ as previously described [51]. n = 6 mice/group. Statistical significance was assessed by log-rank (Mantel-Cox) test

We recently determined that FLU can inhibit melanoma growth as xenografts in immunocompromised mice [14]. To explore whether FLU can influence melanoma growth in immunocompetent animals, we used a tumor isograft model in which FLU was administered systemically (i.p.) to C57BL/6J mice bearing subcutaneous B16F10 tumors and commenced treatment after the tumors were established. The C57BL/6-B16F10 system is a PD-1 antibody resistant mouse model of melanoma growth [13]. Ten days after melanoma inoculation, tumors grew rapidly in the vehicle group (Fig. 1A, B). On the other hand, FLU delayed melanoma growth by several days and caused near cessation of growth after 15 days, with tumors in the vehicle group measuring ~ 1000 mm^3^ while those treated with FLU measuring ~ 200 mm^3^ (Fig. 1B). FLU reduced tumor growth rate between Day 15 to 17 (Fig. 1C). FLU enabled survival in 100% of melanoma bearing mice after 17 days, whereas survival of tumor-bearing mice treated with vehicle after this time was 0% (Fig. 1D).

### ***FLU inhibits PD-1 levels without affecting levels of PD-L1 and stimulates CD3***^+^***cell accumulation***

**Fig. 2:**
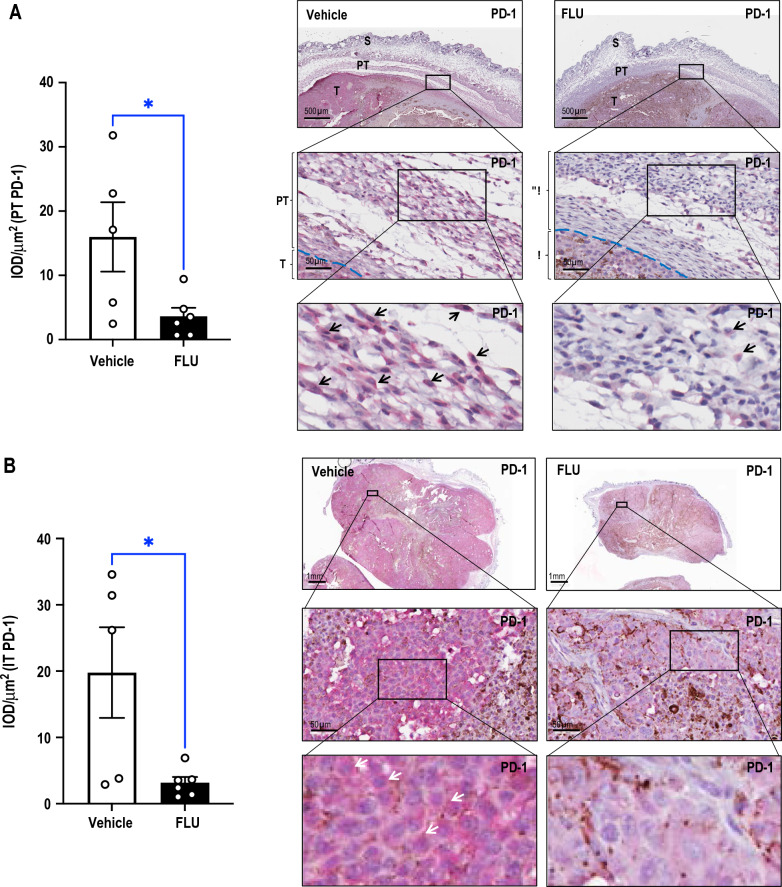

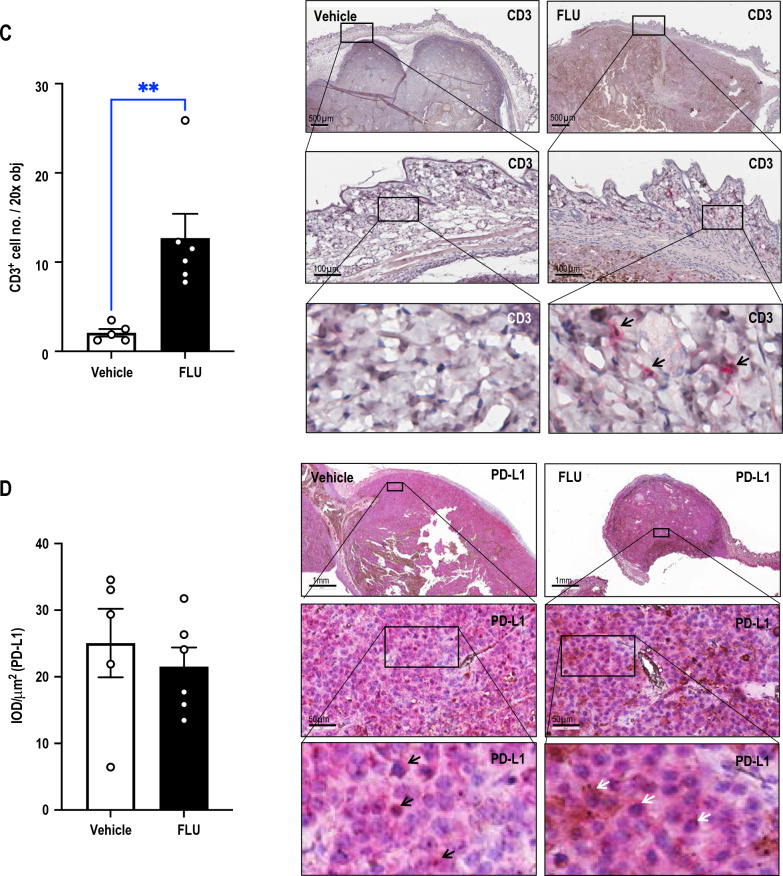
FLU inhibits PD-1 levels without affecting PD-L1 and stimulates CD3^+^ cell accumulation. Immunohistochemical analysis was performed on Day17 B16F10 tumors from vehicle or FLU-treated mice. Quantification and representative photomicrographs of immunostaining for **A** peritumoral, **B** intratumoral PD-1^+^, **C** peritumoral skin CD3^+^ and **D** intratumoral PD-L1^+^. IOD and tissue area were assessed using Image-Pro Plus® and mean of IOD/µm^2^ was determined. Representative immunohistochemical staining is shown (right). Data was analyzed by t-test or Mann Whitney test (n = 5–6 mice/group) (left). Error bars represent SEM. S denotes skin, PT denotes peritumoral area, T denotes tumor, IT denotes intratumoral. Arrows indicate positive staining. Dashed lines separate PT and T

Immunohistochemical staining of the tumors revealed that systemic FLU treatment suppressed PD-1 expression, both peritumorally (Fig. 2A) and intratumorally (Fig. 2B). FLU also caused the dramatic accumulation of CD3^+^ cells as compared with animals treated with vehicle (Fig. 2C). In contrast, FLU treatment did not affect levels of PD-L1 (Fig. 2D).

### *FLU inhibits PD-1 protein expression in Jurkat T cells*

**Fig. 3:**
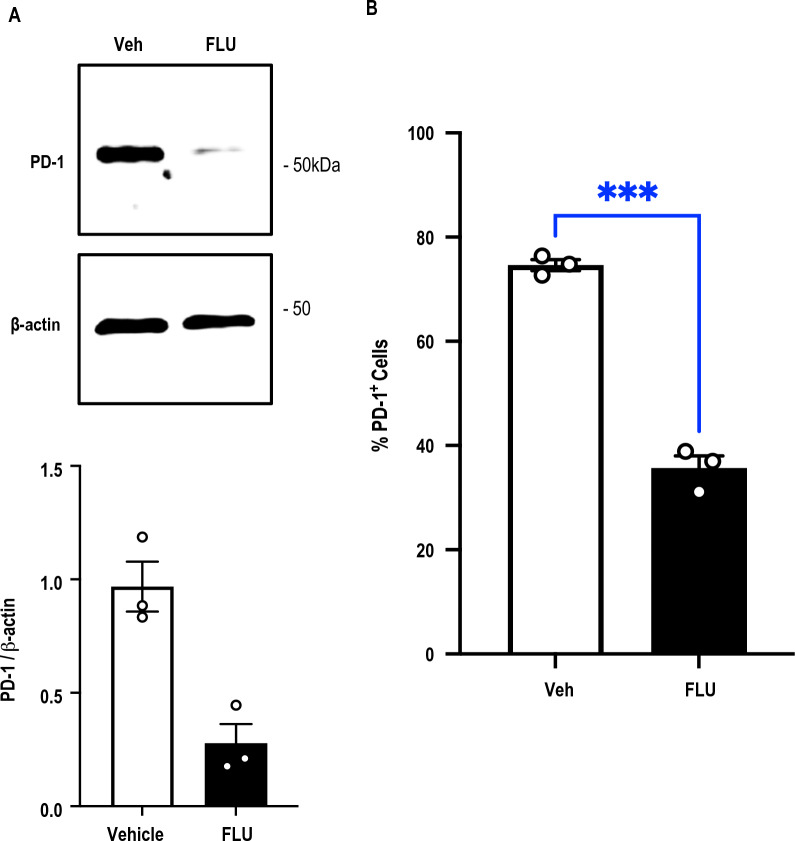
FLU inhibits PD-1 expression in Jurkat T cells. **A** Western blotting was performed with extracts of Jurkat T cells treated with FLU (10 µM) or vehicle for 24 h. Membranes were treated with antibodies to PD-1 or β-actin then with secondary antibodies for detection. **B** Alternatively, cells treated with FLU (10 µM) or vehicle for 24 h were tested for PD-1 immunopositivity by flow cytometry. Data is representative of 3 biologically-independent experiments. Error bars represent SEM. Statistical significance was assessed by t test

Western blotting with extracts of Jurkat cells, a model human T cell line, revealed that FLU reduced PD-1 protein expression within 24 h (Fig. 3A). These findings were supported by separate experiments using flow cytometry (Fig. 3B).

### *RNA-seq reveals that FLU modulates gene expression*

**Fig. 4:**
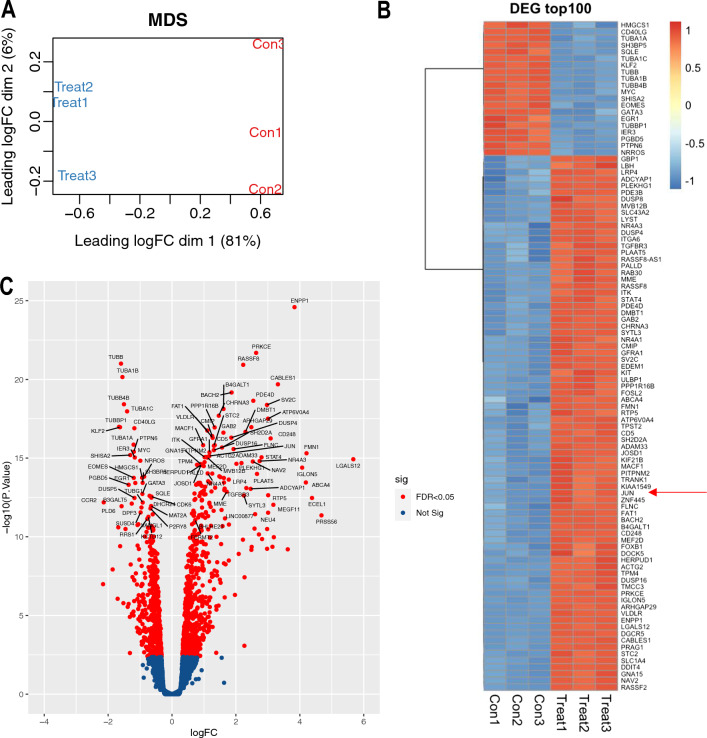
FLU modulates gene expression in Jurkat T cells. RNA-seq was performed with total RNA prepared from Jurkat T cells treated with 10 µM FLU for 4 h. **A** Multidimensional scaling (MDS) analysis. Con1, Con2, Con3 and Treat1, Treat2, Treat3 refer to vehicle and FLU treatment, respectively, where each is a biologically-independent replicate. **B** Heat map showing the top 100 DEG in our analysis. Heatmap was constructed based on z-scores of normalized expression values for the DEG and display expression levels between the biologically-independent samples. Arrow indicates JUN. **C** Volcano plot showing representative genes that down-regulated *(left)* and up-regulated *(right)* by FLU

To gain insights into FLU’s mechanism of action, we exposed Jurkat T cells to FLU or vehicle for 4 h prior to harvest and bulk next generation RNA sequencing (RNA-seq). We chose this early time point to help identify regulatory factors that regulate PD-1 expression controlled by FLU. Multidimensional scaling (MDS) (Fig. 4A**)** showed clear separation between treatment groups (3 biological replicates per group). RNA-seq revealed that from a pool of 14,475 genes (12,259 protein coding, 2216 non-protein coding) (Additional file 1: Table S1) there were 1218 DEG (Additional file 2: Table S2); 687 genes increased expression and 531 reduced expression by FLU with an adjusted p value of < 0.05 (Additional file 2: Table S2). Surprisingly, RNA-seq revealed that at the 4 h timepoint, FLU increased *pdcd-1* (PD-1) mRNA levels by 2.3 fold (Additional file 2: Table S2). Among genes with the largest fold increase in expression with FLU compared to vehicle were galectin 12 (LGALS12, 51.1 fold), serine protease 56 (PRSS56, 25.6 fold), endothelin converting enzyme like 1 (ECEL1, 20.1 fold), formin 1 (FMN1, 18.5 fold) and ecto-nucleotide pyrophosphatase/phosphodiesterase (ENPP1, 14.3 fold) (Additional file 2: Table S2, Fig. 4B, C). On the other hand, genes with the largest fold reduction in expression with FLU were interleukin 26 (IL26, 0.22 fold), c–c chemokine receptor type 2 (CCR2, 0.23 fold), zinc finger and BTB domain containing 16 (ZBTB16, 0.28 fold) and tripartite motif containing 67 (TRIM67, 0.31 fold) (Additional file 2: Table S2, Fig. 4B, C).

**Fig. 5:**
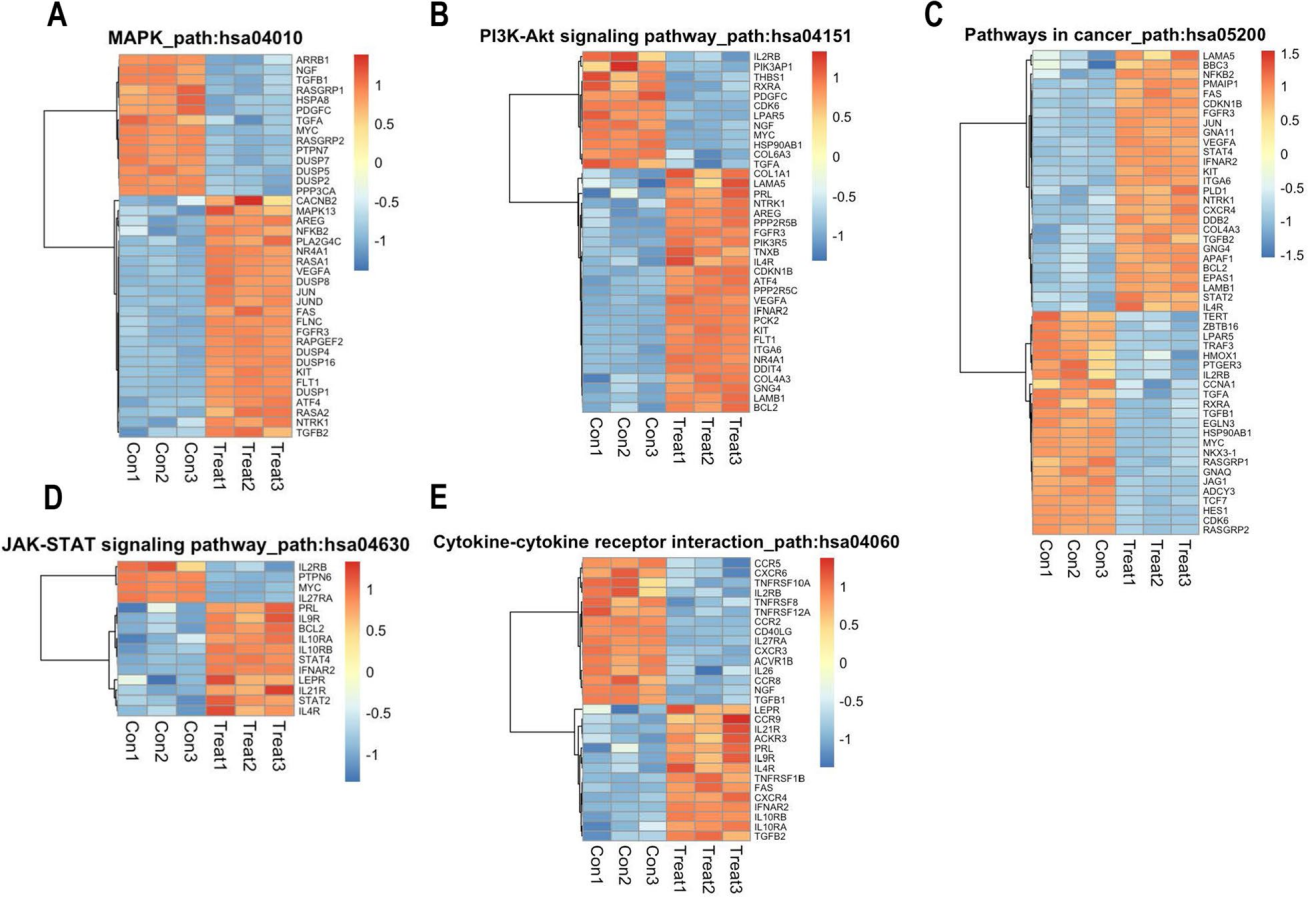
Heat map representation of genes significantly up- or down-regulated by FLU in Jurkat T cells. These genes are associated with the **A** MAPK pathway, **B** PI3K-Akt signaling pathway, **C** pathways in cancer, **D** JAK-STAT signaling pathway, and **E** cytokine-cytokine receptor interactions. RNA-seq was performed with total RNA prepared from Jurkat T cells treated with 10 µM FLU for 4 h. Heatmaps were constructed based on z-scores of normalized expression values for the DEG and display expression levels between the biologically-independent samples. Each row is scaled using the scale function of the heat map tool which takes the z-score across each row

Kyoto Encyclopedia of Genes and Genomes (KEGG) pathway analysis was performed to determine whether DEGs following FLU treatment were over-represented in specific pathways. The up-regulated genes were over-represented in 25 pathways (p < 0.01), the top hit was amoebiasis, not surprising for an anthelmintic. FLU also up-regulated genes in pathways associated with hematopoeisis and PI3K-Akt signaling (Additional file 3: Table S3A and Fig. 5). Pathways associated with cancer, MAPK signaling and cytokine-cytokine receptor interactions were among those which had a significant over-representation in both up-regulated and down-regulated genes (Additional file 3: Tables S3A, B and Fig. 5).

**Fig. 6:**
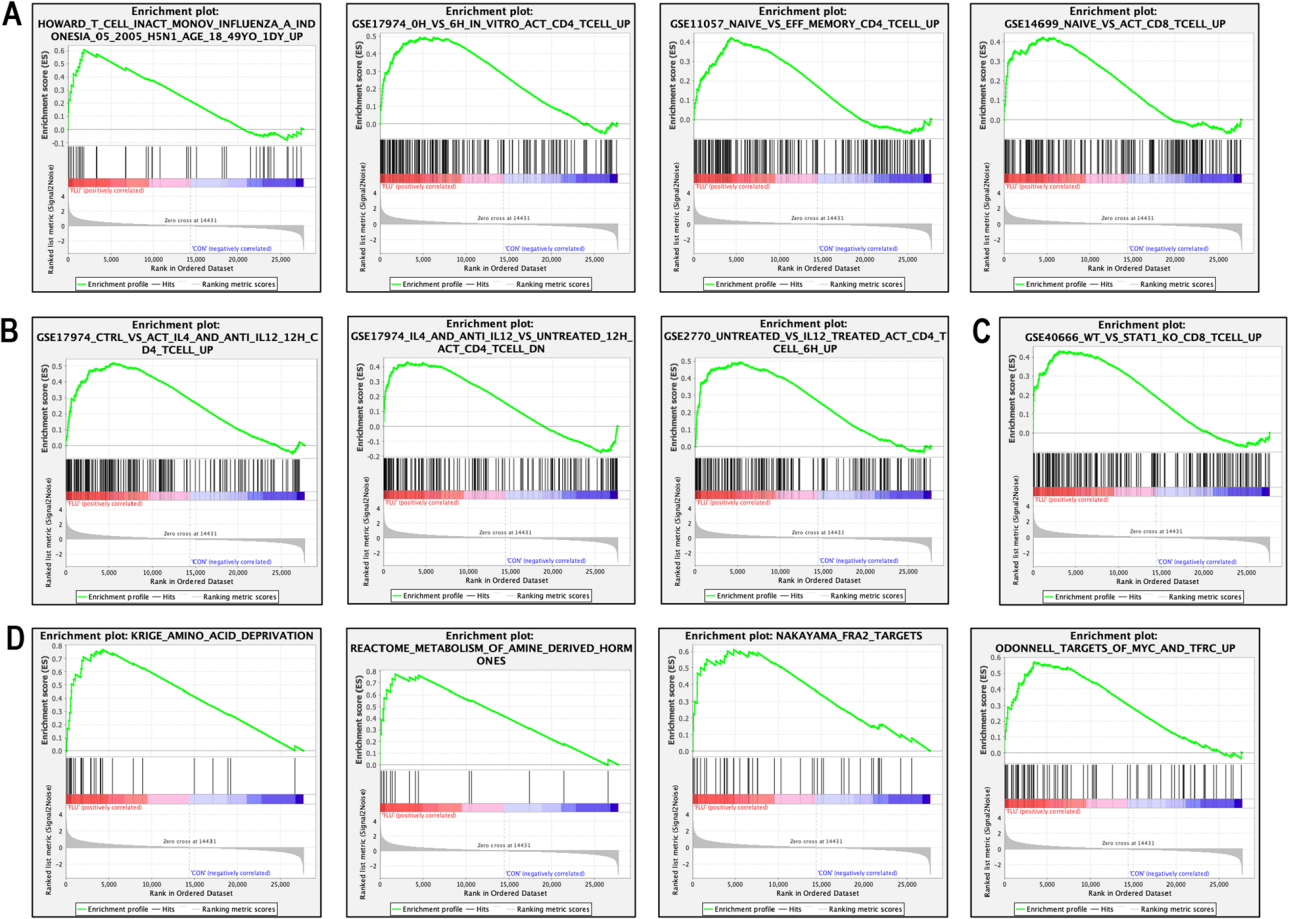
Representative GSEA of gene sets enriched by FLU in Jurkat T cells. Representative C7 gene sets enriched by FLU were those associated with T cell **A** function, **B** differentiation and **C** proliferation. Representative C2 gene sets enriched by FLU were those associated with cell metabolism and cell signaling (**D**)

Given FLU’s effects on PD-1 expression in Jurkat T cells and its ability to induce CD3^+^ T cell accumulation in tumors whose growth it inhibits, we performed GSEA on gene sets in the C7 collection representing cell states and perturbations within the immune system. This identified a large number of immunologic signature gene sets that correlated with FLU treatment (605 sets FDR < 0.25) (Additional file 4: Table S4), including genes associated with T cell function, differentiation and proliferation (Fig. 6A–C). GSEA performed with the C2 collection identified 210 gene sets correlated with FLU treatment, including those associated with cell metabolism and cell signaling (Additional file 5: Table S5 & Fig. 6D) providing further biological insight into the potential actions of FLU.

### *FLU stimulates JUN and FOS expression*

**Fig. 7:**
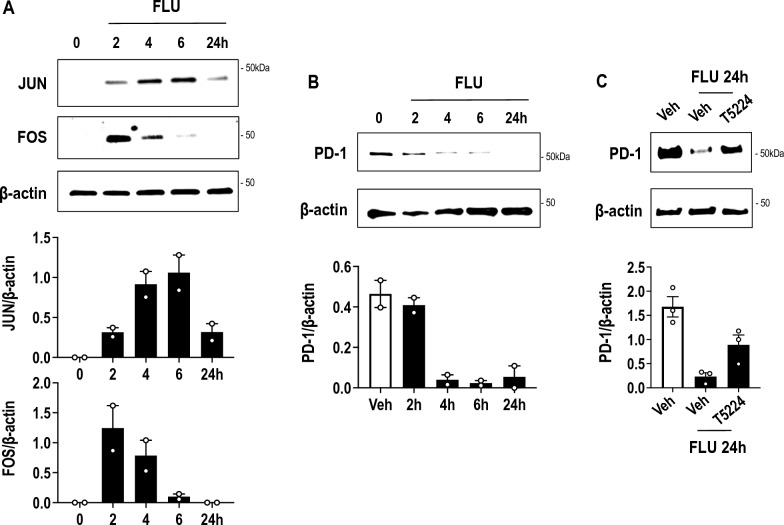
FLU activates JUN and FOS expression in Jurkat T cells and its suppression of PD-1 expression is rescued by T5224. Western blotting was performed with extracts of Jurkat T cells treated with FLU (10 µM) or vehicle for various times. Membranes were treated with antibodies to **A** JUN, FOS or β-actin, and **B** PD-1 or β-actin then with secondary antibodies for detection. **C** Western blotting was performed with antibodies to PD-1 (pretreated with vehicle or 10 µM T5224 for 2 h) then treated with FLU (10 µM) for a further 24 h. Densitometry was performed using NIH Image J. Error bars represent SEM. Data is representative of 3 biologically-independent experiments

FLU caused after 4 h a 2.2 fold increase in mRNA levels of the transcription factor, *JUN* (Additional file 2: Table S2, Fig. 5A, C), a member of the basic leucine zipper (bZIP) transcription factor family. Interestingly, recent studies show that overexpression of *JUN* in T cells can reinvigorate T cells and improve anti-tumor potency [24]. *JUND* and *JUNB* expression was modulated 1.61 and 0.67 fold, respectively, by FLU, while another immediate-early gene *EGR1* [25–28] was modulated 0.45 fold (Additional file 2: Table S2). FLU induction of JUN at the level of protein was confirmed by Western blotting which showed that JUN levels were induced within 2 h and peaked at 6 h (Fig. 7A**)**. This, to the best of our knowledge, is the first demonstration of FLU’s induction of JUN in any cell type. FLU also caused a transient induction of FOS protein, peaking at 2 h, with substantially earlier induction kinetics compared with JUN (Fig. 7A), which may explain why RNA-seq detected increased levels of *JUN* but not *FOS* (Additional file 2: Table S2, Fig. 5A, C). Others have also noted more acute and less sustained induction of FOS protein than JUN [29, 30]. These experiments also showed that FLU inhibited PD-1 protein expression within 2–4 h (Fig. 7B).

Finally we determined whether FLU’s suppression of PD-1 was reliant upon AP-1. We exposed Jurkat T cells to FLU after preincubation with T5224 (3-(5-(4-(cyclopentyloxy)-2-hydroxybenzoyl)-2-((3-hydroxybenzo[d]isoxazol-6-yl)methoxy)phenyl)propanoic acid), a small molecule benzophenone-based selective inhibitor of AP-1 (FOS/JUN) binding to DNA [31, 32]. FLU suppressed PD-1 protein expression which was prevented by T5224 (Fig. 7C) suggesting that PD-1 inhibition by FLU relies, at least in part, upon AP-1.

## Discussion

Immune checkpoint inhibitor therapy has fundamentally changed the clinical management of advanced melanoma and a range of other cancers. Monoclonal antibody blockade of the PD-1/PD-L1 system seeks to re-orientate immunoediting toward immunosurveillance and immune-mediated tumor recognition and lysis. Adjuvant use of immune checkpoint inhibitors in melanoma is now directed by clinical practice guidelines [33]. Recent research has demonstrated survival advantage from neoadjuvant-adjuvant immune checkpoint inhibitor therapy in melanoma, suggesting benefit in supporting the immune system to recognise the tumor prior to excision [34]. Notwithstanding this, ablative surgical intervention remains the primary treatment modality for local or locoregional melanoma given that objective response rates and response durability remain unsatisfactory for most patients. Toxicity remains a concern and dosing require hospital attendance. Repurposing existing therapeutic agents may improve response, durability and offer additional “concurrent” therapies; whilst mitigating adverse events and providing for an “at home” per oral regime. Given the potential for these repurposed agents to target signaling upstream of PD-1/PD-L1, a non-surgical approach to melanoma may be realised.

This study provides the first demonstration that the anti-parasitic drug FLU inhibits melanoma growth in immunocompetent mice with PD-1 inhibition through AP-1. RNA-seq analysis revealed that hundreds of genes were differentially expressed in a human T cell line exposed to FLU. One of these genes was the prototypic AP-1 family member, *JUN*. T5224, a small molecule that blocks AP-1 binding to DNA [31, 32], prevented FLU suppression of PD-1 expression. That FLU inhibits both tumor growth and PD-1 protein expression in immunocompetent mice suggests the potential use of this anthelmintic as a repurposed, small molecule inhibitor for melanoma and possibly other cancer types.

Previous studies have determined that FLU can inhibit breast cancer, gastrointestinal cancer, melanoma, neuroblastoma, oral carcinoma, leukemia and myeloma in mice [18]. However, to the best of our knowledge, the present study is the first to show that FLU can inhibit melanoma growth with suppression of PD-1 in immunocompetent mice and builds upon our earlier demonstration in immunodeficient mice [14]. Our previous study [14] showed that FLU can abrogate growth and metastasis of human melanoma grown as xenografts in SCID mice. FLU inhibited levels of phospho-(Tyr^705^) STAT3, PD-1 and myeloid-derived suppressor cell (MDSC) accumulation within the tumors [14]. On the other hand, the present study investigated the effect of FLU on murine melanoma growth in immunocompetent mice, we found FLU inhibited tumor growth and suppressed PD-1 levels in the tumor microenvironment. While we did not investigate the involvement of MDSC or macrophages, we found that FLU increased immunostaining for the pan T cell marker CD3^+^ and inhibited PD-1 expression in Jurkat cells, a model human T cell line. Moreover, unlike our previous work [14], this study incorporates the results of differential gene expression and bioinformatics analysis which provide new insights on transcriptional changes effected by FLU.

Suppression of peritumoral and intratumoral PD-1 by FLU was accompanied by increased CD3^+^ T cell accumulation in peritumoral skin. Park et al.also observed changes in CD3^+^ T cell populations in peritumoral skin [35], while Haywood et al. and Halse et al. reported changes in peritumoral PD-1 expression [36, 37]. FLU inhibited tumor growth and PD-1 levels in this study without affecting levels of PD-L1. KEGG and GSEA provided novel insight into the potential functions of FLU. For example, FLU up-regulated genes associated with PI3K-Akt signaling, a pathway linked with the development of T cell effector function [38] and reduced regulatory T cell phenotype and metabolic state [39]. This is in line with GSEA that identified a range of immunologic signature gene sets correlated with FLU treatment such as those underpinning T cell function, differentiation and proliferation. Future studies should determine the direct effects of FLU on T cell state and function.

PD-1 expression is regulated at the level of transcription by factors such as signal transducer and activator of transcription (STAT) and members of the AP-1 family [40–42]. AP-1 is induced by TCR signalling, cooperates with NFAT in T cells and drives IL-2 expression and effector function. The present study demonstrates that FLU increases JUN expression and that AP-1 is needed for FLU’s suppression of PD-1. T5224 has been used previously to demonstrate an important role for AP-1 in prolyl-isomerase expression in Jurkat T cells [43] and in pancreatic beta-like cells where it rescued mutant CDKAL1-associated beta cell defects [44]. Effects on PD-1 levels using T5224 and FLU are supported by recent findings by Carnevale et al. showing that increased AP-1 activity prevents T cell exhaustion and/or T cell anergy in Jurkat T cells [45]. Our findings showing differences in *pdcd-1* mRNA and PD-1 protein expression levels at the 4 h timepoint suggest complex regulation. This could arise from altered *pdcd-1* transcription or mRNA stability as a compensatory response to reduced levels of PD-1 protein in cells exposed to FLU. For example, PD-1 can undergo dynamic ubiquitination and proteasomal degradation in T cells [46]. Conversely, inducible FOS protein but not mRNA levels at this timepoint could reflect shorter lived *FOS* mRNA in cells exposed to FLU.

Repurposing FLU as a compound that can stimulate anti-cancer immunity is attractive since the benzimidazole has established safety profiles in humans and animals. FLU has been used for the treatment of gastrointestinal nematode infections in humans and animals since the 1980s. The dose of FLU for use as an anthelmintic in humans (oral 100 mg dose twice a day for ascariasis, trichuriasis, ancylostomiasis, mixed infections) is substantially below the dose used in this cancer study (200 mg/kg), which is that we used previously in immunodeficient mice [14]. Spagnuolo et al. administered FLU at 200 mg/kg daily i.p. for 2 weeks and found that it did not induce neuropathy [47]. FLU is a compound with an extremely high LD_50_ (> 5000 mg/kg) in mice, rats and guinea pigs [48, 49] and is metabolized. In human liver, FLU is reduced in cytosol by carbonyl reduction and NADPH is the preferred coenzyme [50]. This study provides the first evidence that FLU can inhibit tumor growth with reduced PD-1 expression in immunocompetent mice. Our study suggests the potential use of FLU as a repurposed small molecule inhibitor in melanoma and potentially other cancers involving PD-1.

## Supplementary Information

**Additional file 1: Table S1.** Pool of 14475 genes in bioinformatics analysis of RNA-seq data showing 12259 protein coding and 2216 non-protein coding genes. RNA-seq was performed with total RNA prepared from Jurkat T cells treated with 10 µM FLU for 4 h

**Additional file 2: Table S2.** Complete list of DEG following RNA-seq and bioinformatics analysis. RNA-seq was performed with total RNA prepared from Jurkat T cells treated with 10 µM FLU for 4 h.

**Additional file 3: Table S3.** KEGG pathway analysis of RNA-seq data. RNA-seq was performed with total RNA prepared from Jurkat T cells treated with 10 µM FLU for 4 h. Over-representation of DEG following FLU treatment in specific pathways that were (**A**) up-regulated and (**B**) down-regulated

**Additional file 4: Table S4.** C7 gene sets enriched by FLU. RNA-seq was performed with total RNA prepared from Jurkat T cells treated with 10 µM FLU for 4 h. There were 3738 of 5082 C7 gene sets upregulated in the FLU group, 605 sets significant at FDR < 0.25, and 1344 of the 5082 gene sets correlated with the vehicle, 381 were significant at FDR < 0.25.

**Additional file 5: Table S5.** C2 gene sets enriched by FLU. RNA-seq was performed with total RNA prepared from Jurkat T cells treated with 10 µM FLU for 4 h. There were 3058 of 4691 C2 gene sets upregulated in the FLU group, 210 sets significant at FDR < 0.25, and 1633 of the 4691 gene sets correlated with the vehicle group, 345 sets significant at FDR < 0.25.

## Data Availability

The datasets supporting the conclusions of this article are included within the article.
